# Starches of two water yam (*Dioscorea alata)* varieties used as congeals in yogurt production

**DOI:** 10.1002/fsn3.941

**Published:** 2019-02-10

**Authors:** Charles Tortoe, Paa T. Akonor, Jemima Ofori

**Affiliations:** ^1^ Food Research Technology Division Council for Scientific and Industrial Research ‐ Food Research Institute Accra Ghana

**Keywords:** congeal, *Dioscorea alata*, starch, yogurt

## Abstract

The physicochemical properties of water yam (*Dioscorea alata var. Akaba* and *Matches)* starches were determined prior to their use as congeals for yogurt production. The moisture content ranged from 9.34% to 15.8% for A100 (100% *Akaba*) and M100 (100% *Matches*), respectively, indicating lower moisture content in the *Akaba* variety compared to *Matches* variety. Similar trend was observed for their water activity. The pH ranged from 5.88 to 6.93 indicating low acidity of the water yam starches. The water absorption capacity (WAC) ranged from 4.10 to 4.89 g/g, seemingly restricted reflecting protein–moisture interaction of the starches. Although the swelling power did not differ significantly (*p *>* *0.05) ranging from 10% to 14%, they were quite restrictive as the WAC. The L* values of the starches were predominantly lightness in color, highest for A100 sample. The pasting temperatures of *Akaba* (A100), *Matches* (M100), and A50:M50 were not significantly different (*p *>* *0.05). Peak viscosity of the water yam starches was in a range of 509–528 BU. The highest attributes were for taste (6.4), mouthfeel (5.4), flavor (5.4) sourness (4.6) and consistency (5.9), which were obtained from 1.5 % *Matches*, 0.5 % *Akaba* + 0.5 % *Matches*, 1.0 % *Akaba* + 1.0 % *Matches* samples. The overall acceptability (5.8) was higher than the control yogurt (4.7), indicating sample 0.5% *Akaba *+ 0.5% *Matches* as the best‐bet yogurt.

## 1 INTRODUCTION

Yogurt is a food product of milk caused by bacterial fermentation of the milk. Among the fermented products in the world, yogurt is the oldest, safe, most popular fermented milk product in the world. This is attributed to its taste, perceived therapeutic activity, and high nutritive value (Coïsson, Travaglia, Piana, Capasso, & Arlorio, 2005). Different types of milk are used in the production of yogurt from cow, goat, sheep, mare, camel, and female yak and each one of them has its unique flavors. Although various types of milk can be used to produce yogurt, cow's milk is the most common milk used (Lahtinen, Ourwerhand, Salminen, & Wright, 2012). Plain yogurt from whole milk contains approximately 88% water, 3.5 g protein, 3.3 g fat, 4.7 g carbohydrates, 4.7 g sugar per 100 g, and a pH value of 3.8–4.6 (Tamine, 2002; USDA, 2013). A 100 g yogurt provides 406 KJ (97 Kcal) of dietary energy. According to El‐Abbadi, Dao, and Meydani (2014), a serving of yogurt is a rich source of vitamin B12 (31% DV) and riboflavin (23%) with moderate content of protein, phosphorus, and selenium (14%–19%) as a proportion of the daily value (DV). Yogurt is often associated with probiotics having positive effects on immune, cardiovascular, or metabolic health of humans. It has been found to contain protein, vitamins, a rich source of calcium, acts as a digestive aid, encourages the growth of beneficial bacteria and inhibits the growth of harmful bacteria in the gut, stimulates intestinal immunity and is an excellent food for lactose intolerant people (El‐Abbadi et al., 2014).

Yogurt is produced using a starter bacteria culture of *Lactobacillus delbrueckii* spp *bulgaricus* and *Streptococcus thermophiles* (Sahan, Yasar, & Hayaloglu, 2008; Serra, Trujillo, Guamis, & Ferragut, 2009). In addition, other lactobacilli and bifidobacteria are also sometimes added during or after culturing yogurt. However, due to the expanding regime of functional foods and nutritional needs often accompanying species of live lactic acid bacteria and bioactive compounds, the composition of bacteria starter culture varies at industrial production (Birollo, Reinheimer, & Vinderola, 2000; Park et al., 2005). The fermentation of lactose by these bacteria produces lactic acid, which acts on milk protein to give yogurt its texture and characteristic flavor (Lahtinen et al., 2012). The fermentation process results in partial hydrolysis of fat, protein, and lactose resulting in yogurt been easily digestible compared to milk and suitable for people suffering from lactose intolerance.

Congeals have been added to yogurts to improve the texture and consistency especially without substantially changing its other properties whenever yogurt production end result was thin instead of thick yogurt. Food congeals are based on either polysaccharide (starch, vegetable gums, and pectin) or proteins. Different congeals may be more or less suitable in a given application, due to differences in taste, clarity, and their responses to chemical and physical conditions (Deven, Glassburn, Jodelle, & Deem, 1998). Subsequently, flavorless powdered starches such as arrowroot starch, cornstarch, potato starch, cassava and yam and their derivatives have been used as congeals. Food congeals from vegetable gums included alginin, guar gum, locust bean gum, and xanthan gum. Proteins used as food thickeners included collage, egg white, and gelatin, whereas sugar included agar and carrageenan (Deven et al., 1998). Generally, starch congeals in yogurt improve the viscosity, texture, and mouthfeel and prevent wheying‐off. Starch congeals are popular due to their advantage to thicken yogurts without adding fat and give the food a transparent, glistening sheen, creamy texture as well as ease processing at a lower cost compared to other hydrocolloids (Koegh & O'Kenedy, 1998). Starch granules imbibe water and swell to many times their original size, resulting in increased viscosity of the solution (Basim, Hazim, & Ammar, 2004). The gelatinization results in changes of the granular structure, swelling and hydration, and solubilization of starch molecules. Swelling is accompanied by leaching of granule constituents, mostly amylose. In a mixture of milk and starch, during heat, treatment may lead to different rheological characteristics in the final yogurt gel product compared to that made from milk alone (Narpinder, Jaspreet, Lovedeep, Navdeep, & Balmeet, 2003). Starch behavior in a system like that one of yogurt will also depend on their physical and chemical characteristics, such as mean granule size distribution, amylase/amylopectin ratio, and mineral content.

Yam *(Dioscorea* spp.) is a tropical tuber crop and a major source of income for farmers and traders in sub‐Sahara Africa (Tortoe, Dowuona, Akonor, & Dziedzoave, 2017). Yam is not only a good source of starch and vitamin C, but it is also an important sociocultural crop that is prominent in the cultural and religious festivals of the people of West Africa (Deutsche Gesellschaft fur Technische Zusammenarbeit, 1995). There are more than 200 species of yam in cultivation (Amusa, Adegbite, Mohammed, & Baiyewu, 2003). Water yam (*Dioscorea alata)* is noted for its bulkiness, high moisture, and starch content, although underutilized compared to the popular variety of *Dioscorea rotundata*. Alternative food uses of water yam as a source of suitable congeal in yogurt production would improve its cultivation and increase incomes for farmers and traders and expand its food forms for consumers. In expanding the food uses of *Dioscorea alata,* this study was aimed at analyzing some physicochemical properties of starches obtained from two varieties of *Dioscorea alata* (*Akaba* and *Matches*) in Ghana prior to evaluating their sensory acceptability as congeals in yogurt production.

## 2 MATERIALS AND METHODS

### 2.1 Raw materials

Matured water yam (*Dioscorea alata* var *Akaba* and *Matches*) were obtained directly from farms in Atebubu‐Amantin District of Brong Ahafo and identified by Extension Officers of the Ministry of Food and Agriculture (MoFA) in Atebubu, Ghana.

### 2.2 Natural starch extraction

The matured water yam varieties (*Dioscorea alata* var. *Akaba* and *Matches)* were washed under running water, peeled with a stainless steel knife, and washed again. The peeled tubes were immediately cut into thin slices (5 mm) into a plastic basin containing a solution of 1% sodium metabisulfite. The slices were removed after 10 min with a sieve to allow adhering water to drain and blended into a slurry using a blender (Phillips 8010G, USA). Hundred grams (100 g) of yam slices were blended with 200 g distilled water. The slurry was filtered through a clean cheesecloth. The mixture was allowed to sediment. The filtrate was decanted. Subsequently, the sediment was mixed with distilled water, allowed to sediment and the filtrate decanted. The washing process was repeated three times until there was little or no starch in the filtrate. The sediment was spread thinly on drying trays and dried in a mechanical dryer (CSIR‐Food Research Institute, Accra) at 40°C for 5 hr. The dried starch was milled using a disk attrition mill (Premier No.2, India) and the starch packaged airtight in high‐density polyethylene bags until use. The formulations of the water yam starches used in the study are presented in Table 1.

**Table 1:** Formulation of water yam starches

Sample	Formulation	Water yam variety starch (%)	
		*Akaba*	*Matches*
A	A100	100	0
B	M100	0	100
C	A10M90	10	90
D	A20M80	20	80
E	A30M70	30	70
F	A40M60	40	60
G	A50M50	60	50
H	A60M40	60	40
I	A70M30	70	30
J	A80M20	80	20
K	A90M10	90	10

Note

A100: 100% *Akaba*; A10M90: 10% *Akaba *+ 90% *Matches*; M100: 100% *Matches*.

### 2.3 Determination moisture content

Five grams (5.0 g) of starch was measured into an Electronic Moisture Analyzer—Sartorius MA 45 (Sartorius GMBH, Gottingen, Germany) to measure the moisture content. The analysis was performed in triplicates.

### 2.4 Determination of water activity

Using a Rotronic HygroLab 2 (Rotronic AG, Bassersdrof, Germany), the water activity (*a*_w_) of the starch was measured in triplicates. The *a*_w_ was calibrated using a saturated salt solution of relative humidity of 70% (0.75 *a*_w_). In measuring the *a*_w_, 8.0 g of starch was weighed using Sartorius Portable, PT600, Sartorius GMBH, Gottingen, Germany, scale and transferred into the chamber of the Rotronic HygroLab 2. The *a*_w_ was conducted in triplicates.

### 2.5 Determination of pH

The pH of starches was determined using approved methods of the Association of Official Analytical Chemists (AOAC, 2000) in triplicates.

### 2.6 Water absorption capacity

The water absorption capacity (WAC) of the starch was determined by a modified method of Afoakwa, Budu, Asiedu, Chiwona‐Karltun, and Nyirenda (2012). Briefly, 1.0 g of starch was mixed with 10 ml of distilled water for 30 s. The sample was allowed to stand at room temperature (28°C) for 30 min, after which they were centrifuged at 1,050 *g* (Hermle Z 206A, Germany) for 30 min. The volume of the supernatant was recorded and the water absorption capacity calculated as the difference between the initial volume of water added to the starch and the volume of the supernatant. This was done in triplicates and mean values calculated.

### 2.7 Swelling power

Starch dispersions of 2.5% were put in centrifuge tubes, capped to prevent spillage, and heated in a water bath with a shaker (GRANT OLS 200) at 85°C for 30 min. After heating, the tubes were cooled to room temperature and centrifuged at 560 *g* (Remi Research, R23, Germany) for 15 min. The precipitated paste was separated from the supernatant and weighed (*W*_*p*_). The supernatant was evaporated in a hot air oven (Gallenkamp Hotbox, England) at 105°C and the residue weighed (*W*_*r*_). Determinations were done in triplicates, and swelling power (SP) was calculated as:

$$\text{SP}=\frac{\text{wt of precipitated paste}(W_{p})}{\text{wt of the sample}(W_{o})}-\text{wt of residue in the supernatant}\left(W_{r}\right)$$

where *W*_*o*_ is the weight of the sample

### 2.8 Colorimetry

Color analysis of the starch was done in triplicates using a Minolta Chromameter (CR‐310 Minolta, Japan) in triplicates. A reference white porcelain tile (*L*_0_ = 97.63, *a*_0_ = 0.31 and *b*_0_ = 4.63) was used to calibrate the Chromameter before each determination. The starch color was described in L* a* b* notation, where L* is a measure of lightness, a* defines components on the red–green axis, and b* defines components on the yellow–blue axis.

### 2.9 Starch pasting profile

The pasting properties of the starches were determined at 8% slurry using a Brabender Viscoamylograph (Viskograph‐E, Brabender Instrument Inc., Duisburg, Germany) equipped with a 1,000 cmg sensitivity cartridge. The determinations were done for 100% *Akaba,* 100% *Matches,* and a combination of 50% *Akaba*: 50% *Matches* starches. The suspension was heated from 50 to 95°C at a rate of 1.5°C/min, held at this temperature for 15 min, cooled to 50°C at a rate of 1.5°C/min, and held at this temperature for 15 min. The viscosity profile indices recorded included the following: pasting temperature, peak viscosity, viscosity at 95°C and viscosity after 15 min hold at 50°C (50°C‐hold), breakdown and setback.

### 2.10 Preparation of yogurt

The methods described by Lee and Lucey (2010) were modified by homogenizing powdered cow's milk and water and heating at 85–90°C for 5 min in a boiling water bath during which sugar was added at a rate of 6.5% (w/v) to the mixture of custard‐like consistency to denature the milk proteins to avoid the formation of curds. The water yam starch was added to the mixture during the heating. The heated milk and starch mixture was allowed to cool to 45°C and inoculated with 2.5% (w/v) of *Lactobacillus bulgaricus* and *Streptococcus thermophilus* bacteria in a ratio of 1:1 at 45°C. The heated milk and starch mixture was incubated at 42–45°C for 4–5 hr to allow fermentation and setting of the yogurt until a pH of 4.0–4.6 was obtained (Chandan & Kilara, 2010). Nine dairy yogurt samples with different percentages of water yam starch added were *Akaba* variety (0.5%, 1.0%, 1.5%); *Matches* variety (0.5%, 1.0%, 1.5%); and a combination of *Akaba* plus *Match* varieties (0.5%, 1.0%, 1.5%). The prepared yogurts were refrigerated at 4°C until subsequently used. The control sample was a popular full milk dairy yogurt purchased from a supermarket in Accra.

### 2.11 Sensory evaluation of yogurt

A sensory panel consisting of 20 semi‐trained panelists who were familiar with sensory attributes of yogurt was assembled to assess the yogurts. A 7‐point Hedonic scale was used to rate the yogurt for taste, mouthfeel, flavor, sourness, viscosity, and overall acceptability. A score of 1 represented “dislike extremely” and a score of 7 represented “like extremely” (Lawless & Heymann, 2010). Panelists were also given the option to make general comments about the yogurt samples. An atmosphere of complete quietness and privacy was provided for each panelist. The sensory panel judges were placed in a designated sensory facility with individual booths at CSIR‐FRI. A randomized design matrix (XLSTAT 2012, Statsoft, France) was used to code the samples (Hooda & Jood, 2005). The sensory evaluation was conducted at midmorning between 10:30 a.m. and 11:30 a.m. Three samples were evaluated at a time, and panelists were instructed to clean their mouths with a piece of unsalted cracker and rinse with water before tasting subsequent samples. Panelist individual scores were averaged, and the data analyzed using SPSS 17.0.1 (2008). Statistical significance was set at a level of 95% confidence interval.

### 2.12 Data analysis

Data were analyzed for differences using ANOVA, and differences separated by Duncan's multiple range tests (SPSS 17.0.1, SPSS Inc. USA). Statistical significance was set at a level of 95% confidence interval. Results were reported as means ± *SE*, and descriptive results plotted out in the form of bar charts.

## 3 RESULTS AND DISCUSSIONS

### 3.1 Moisture content, water activity, and pH of water yam starches

**Figure 1:**
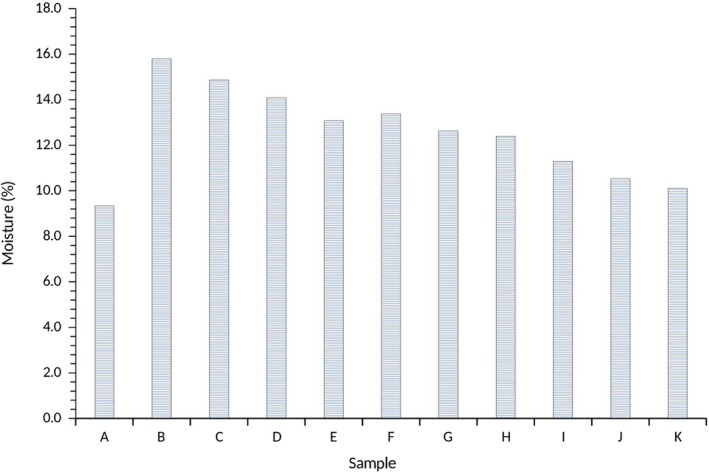
Moisture content of water yam starches

The moisture contents of the water yam starches are presented in Figure 1. The moisture content ranged from 9.34% to 15.8% for A100 (100% *Akaba*) and M100 (100% *Matches*), respectively, indicating lower moisture content in the *Akaba* variety compared to *Matches* variety. In samples consisting of combinations of *Akaba* and *Matches* starches, the moisture content ranged from 10.11% to 14.87% for A90: M10 and A10: M90, respectively. According to van Hal (2000), moisture content corresponds directly to the drying method and time as well as storage conditions. Therefore, lower moisture content supports the shelf‐life stability of the developed yam starches as it prevents the growth of microorganisms and further reduces the incidence of physical and chemical reactions that are likely to cause deterioration and lower the starch quality. Similar observations were reported in the studies of van Hal (2000) and Aguilera, Del Valle, and Karel (1995). Interestingly, the lowest moisture content was observed in A100 (100% *Akaba*), followed by A90:M10 (a combination of 90% *Akaba* and 10% *Matches* yam starch).

**Figure 2:**
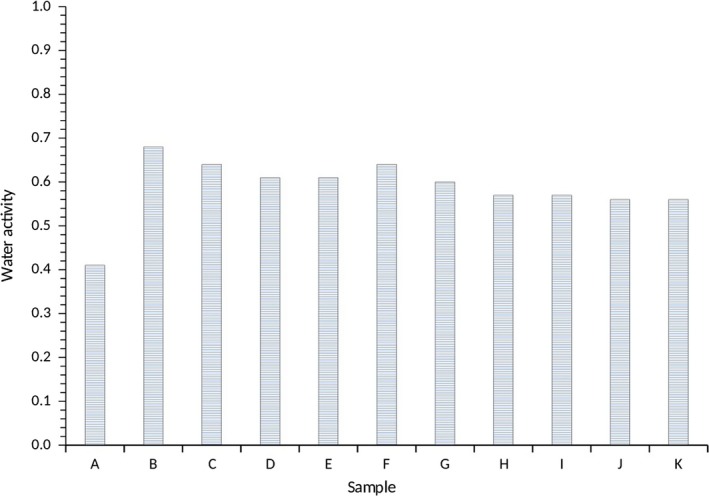
Water activity content of water yam starches

The water activity was lowest in A100 (0.41) and highest in M100 corresponding to the similar trend observed in the moisture content. The water activity of samples of yam starches consisting of *Akaba* and *Matches* combinations was in the range of 0.56–0.64 for A90:M10 and A10:M90 (Figure 2). Both the water activity and the moisture contents have an important influence on the storage properties and quality of the water yam starches as they can enhance the physical and biochemical reactions and also support the growth of microbial organisms, which result in product spoilage and subsequent loss of quality. Interestingly, the moisture content and the water activity of the water yam starches significantly differed (*p *<* *0.05) between the samples (Figures 1 and 2), although they were lower to extend the shelf life of the water yam starches (CAC, 1985). Other studies on starches reported similar trends of 10–12% for millet, corn, and cocoyam starches, which were considered to be within the acceptable range and beneficial in terms of storage and keeping quality of the starches (Mepba, Eboh, Eko, & Ukpabi, 2009; Suma & Urooj, 2015). According to Aguilera et al. (1995), high amounts of moisture in flour and starches may results in caking due to aggregation of particles into lumps, which subsequently lowers their quality and functionality.

**Figure 3:**
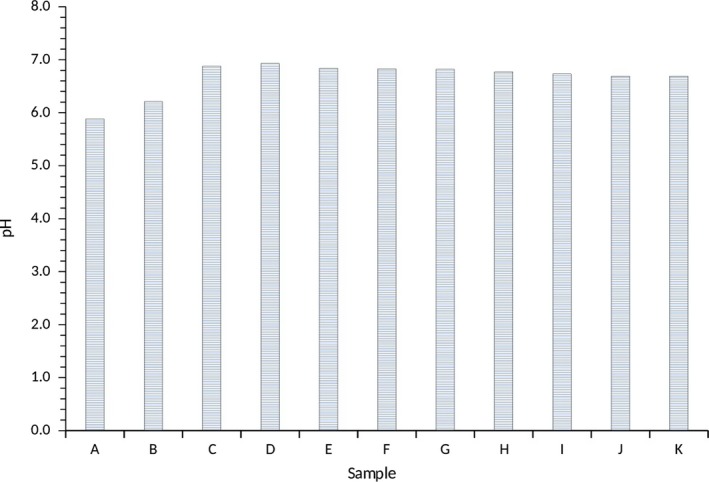
pH of water yam starches

The pH, which is also an indicator of the starch quality, ranged from 5.88 to 6.93 for A100 as the lowest and A10:M90 as the highest (Figure 3). Changes in the pH may affect the functionality of the water yam starches. Samples with a combination of *Akaba* and *Matches* water yam starches had an average pH range of 6.69–6.88 for A20:M80 and differ significantly (*p *<* *0.05). The pH range suggested that the water yam starches are a low acid commodity (Thomas & Atwell, 1999) comparable to cocoyam and cassava starches. Generally, low acidic starches are necessary for the food manufacturing industry.

### 3.2 Water absorption capacity and swelling power

**Figure 4:**
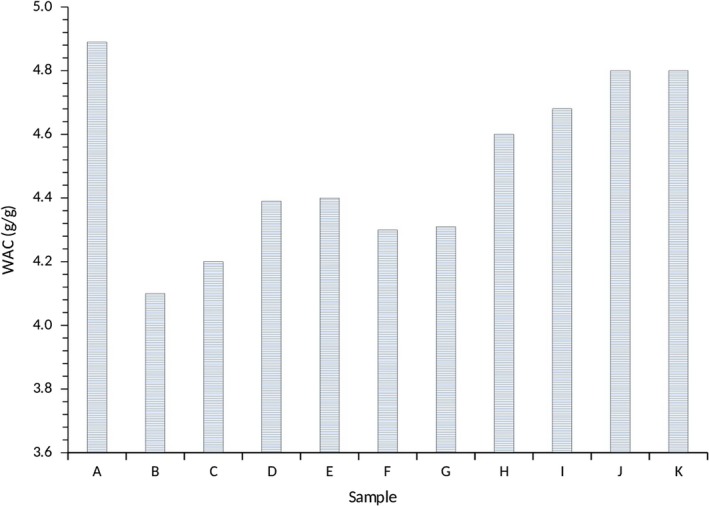
Water absorption capacity of water yam starches

The water yam starches were seemingly restricted in their water absorption capacity (WAC), which was observed in a range of 4.10–4.89 g/g for M100 as the lowest and A100 as the highest (Figure 4). Clearly, the WAC conforms to the moisture content and the water activity as observed for M100 with highest moisture content (15.8%) and highest water activity (0.68), whereas A100 had the lowest moisture content (9.34%) and lowest water activity (0.41; Figures 1 and 2). This indicated that the low moisture content and water activity of the samples had a high affinity for water, thus resulting in the highest WAC for A100 water yam starch. The WAC of the samples with a combination of *Akaba* and *Matches* starches was in the range of 4.20–4.80 g/g. The water yam starches were significantly different (*p *<* *0.05) in their WAC behavior and were lower compared to studies reported by Bhupender, Rajneesh, and Baljeet (2013). Interestingly, the WAC observed was lower than that observed by Osundahunsi, Fagbemi, Kesselman, and Shimoni (2003). The WAC is a reflection of the protein–moisture interaction of the yam starches. Water yam starches with high amounts of proteins possess a lot of water‐binding sites, which increases their WAC as observed by other authors (Bhupender et al., 2013). In other studies, the high WAC was attributed to the loosely associated amylose and amylopectin and the association of hydroxyl groups to form hydrogen and covalent bonds between starch chains (Das, Singh, Singh, & Riar, 2010). Generally, differences in WAC may be attributed to differences in starch structure and morphology, amylose and amylopectin and the presence of salts, proteins and other granular components brought about by differences in genetic makeup.

**Figure 5:**
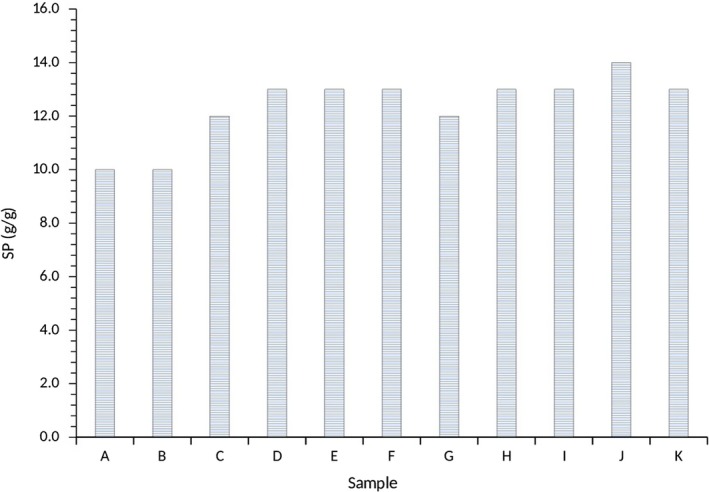
Swelling power of water yam starches

The swelling power (SP) of the water yam starches was in the range of 10%–14%, quite restrictive and did not differ significantly (*p *>* *0.05). The lowest SP (10%) was recorded for A100 and M100, whereas the highest (14) was observed for A80:M20 (Figure 5). Swelling power is influenced by amylose and amylopectin content as well as their chain length distribution resulting in the similarities among the water yam starches observed. The SP is indicative of an intermolecular association between starches polymers associated with eating quality and is influenced by amylose that acts as both diluent and an inhibitor of swelling, which is responsible for retrogradation, whereas amylopectin is responsible for gelatinization behavior of starches (Tester & Morrison, 1990). It measures the hydration capacity of starches and is temperature dependent and accompanied by solubilization of starch granule constituents (Dorporto, Mugridge, Garcia, & Vina, 2011). The SP observed in this study was similar to that obtained for nonirradiated sweet potato starches as reported by Srichuwong, Sunarti, Mishima, Isono, and Hisamatsu (2005) and Ocloo, Bansa, Boatin, Adom, and Agbemavor (2010). In studies by Swinkles (1985), the author reported that yam bean starch had lower swelling power than cassava starch. The highest swelling power obtained for A80:M20 indicated the presence of high amylose content and probably indicate synergetic interactions between the different starches. Further, Sanni, Ikuomola, and Sanni (2001) reported that the high SP in potato was due to the high phosphate content that allows easier water entrance into the granules. According to Lindeboom, Chang, and Tyler (2004), amylose and amylopectin content are responsible for the properties of starch pastes, gels, and starchy food systems. Additionally, amylopectin is either short or long chains and starches with higher amounts of long chains result in gels with higher viscosity and stability compared to the short chain counterparts according to Wrolstad and Smith (2010).

### 3.3 Color

**Figure 6:**
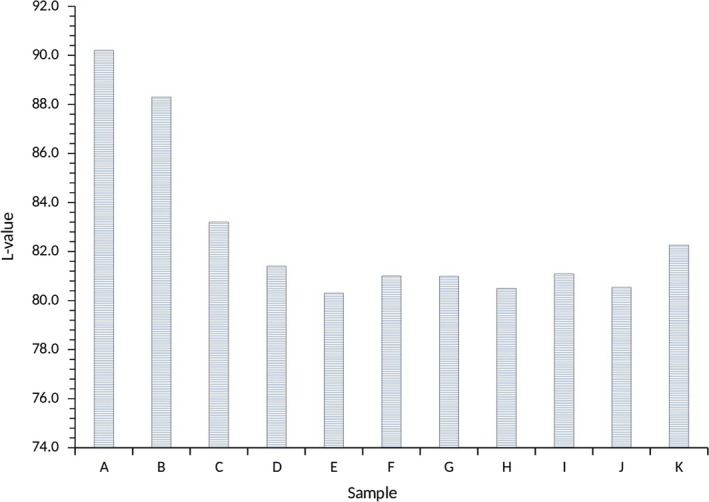
Color (*L*‐value) of water yam starches

The water yam starches L* values were in a range of 80.30–90.20 (Figure 6). The highest L* value was observed for A100 and M100 of 90.2 and 88.3, respectively. The samples of combinations of *Akaba* and *Matches* were in the range of 83.2–80.3. The L* value is indicative of the lightness or darkness of the water yam starches. The darkness was due to extent of browning, which may have occurred during the processing of the water yam starches (van Hal, 2000). The lightness color (90.2) of sample A100 was similar to 91.87 for wheat flour but differs from sweet potato flour (86.62). Color is an important parameter in the choice of food (van Hal, 2000; Wrolstad & Smith, 2010).

### 3.4 Pasting properties of the starches

The pasting properties of the best‐bet water yam starches are presented in Table 2. The pasting temperatures of *Akaba* (A100), *Matches* (M100), and A50:M50 were similar without significant differences (*p *>* *0.05) between them, which indicated that the swelling of the water yam starch granules commenced at similar temperature when subjected to heat (Table 2). This results in the formation of a viscous paste (Afoakwa, Adjonu, & Asomaning, 2010). The pasting temperature observed in this study was higher than that of yam bean starch paste reported in a range of 53–63°C, which was similar to that for cassava and sweet potato (Aprianita, 2010). However, the peak viscosity of the water yam starches in a range of 509–528 BU was lower than that reported by Sefa‐Dedeh and Sackey (2002) for red variety cocoyam starch in a range of 930–1,370 BU. The peak viscosity was highest for A100 sample. The pasting properties aids in the prediction of starch behavior during and after cooking. At the high peak viscosity of 95°C, there was a slight reduction in viscosity with the highest drop in torque observed for A50:M50 sample indicating low shear stability and reduced viscosity after reaching the maximum value 95°C, similarly reported by Singh, Singh, Kaur, Sodhi, and Gill (2003). The cool paste viscosity at 50°C showed the highest viscosity in a range of 635–669 BU and a breakdown in a range of 8–15 BU, simply reflects the resistance to stirring of the swollen mass gel particles due to the presence of amylose fraction responsible for the structure and pasting behavior water yam starch.

**Table 2:** Pasting properties of starches from water yam (*Akaba* and *Matches*)

Sample	Formulation	PT (°C)	PV (BU)	HPV (BU)	CPV (BU)	BD (BU)	SB (BU)
A	A100	81.1 ± 1.0^a^	528 ± 2^a^	517 ± 1^a^	669 ± 3^c^	11 ± 2^ab^	152 ± 2^b^
B	M100	83.3 ± 1.3^a^	509 ± 2^b^	499 ± 4^b^	635 ± 3^a^	8 ± 2^a^	136 ± 3^a^
G	A50:M50	82.9 ± 1.0^a^	520 ± 2^c^	505 ± 3^b^	653 ± 1^b^	15 ± 2^b^	148 ± 3^b^

Note

Means bearing different superscripts are significantly different (*p *<* *0.05).

BD: breakdown; CPV: cool paste viscosity at 50°C; HPV: hot paste viscosity at 95°C; PT: pasting temperature; PV: peak viscosity; SB: setback.

A higher value breakdown in viscosity results in the lower ability of the sample to withstand heating and shear stress during cooking. The highest breakdown of the water yam starches was 15.0 BU similar to taro flour (15.3 BU) and sweet potato flour (23.40 BU), showing that the water yam starches were able to withstand more heating and shear stress (Shimelis, Meaza, & Rakshit, 2006). The setback was in the range of 136–152 BU. These values indicated that the retrogradation tendency of water yam starches was mainly dominated by amylose gelation. Additionally, the high setback has been associated with a high degree of affinity among starch molecules caused by hydrogen bonding (Sefa‐Dedeh & Sackey, 2002). Generally, the setback viscosities significantly differed between the water yam starches. In other studies, the pasting viscosities were positively correlated and higher pasting viscosities corresponds to higher swelling power and higher water holding capacity of the samples (Singh et al., 2003).

### 3.5 Sensory evaluation of developed water yam starch yogurt

The sensory profiles of the developed water yam yogurt are presented in Table 3. The attribute scores range for taste (3.3–6.4), mouthfeel (3.8–5.4), flavor (3.7–5.4), sourness (3.8–4.6), consistency (4.7–5.9), and overall acceptability (4.2–5.8). All attributes scored highest compared to total score (7.0), except the taste (3.3) of 1.5% *Akaba* sample. This indicated that the water yam starches were completely solubilized in the milk, producing smooth and viscous yogurts. According to Oh, Anema, Wong, Pinder, and Hemar (2006), this was due to starch granules embedding in the continuous protein network during the milk heating process before the fermentation commenced. The gelatinized starch granules led to the formation of large protein particles which gave a thick consistency (Lucey & Singh, 1998). Additionally, the thick consistency was enhanced by the higher serum viscosity as a result of starch constituents leaching out during starch gelatinization, which increased the viscosity of the aqueous phase and subsequently results in a thick smooth consistency yogurt generally acceptable (Narpinder et al., 2003; Oh et al., 2006). Interestingly, during yogurt production, the bacterial strains convert part of the lactose into lactic acid after inoculation. Coagulation of the milk occurs after a sufficient quantity of lactic acid is produced and that affects the characteristic organoleptic properties of the prepared yogurt. The more viable bacteria present in the yogurt determine the freshness the yogurt. The highest attribute for taste (6.4), mouthfeel (5.4), and sourness (4.6) was recorded for 1.5% *Matches* only samples, which were higher than the control yogurt sample (4.0, 4.7, and 3.9). The sample 1.0% *Akaba *+ 1.0% *Matches* scored the highest consistency (5.9) than the consistency of the control yogurt sample (5.3). The overall acceptability highest score of the developed yogurts was 5.8, comparable to the maximum score of 7.0 and higher than the control yogurt sample (4.7). Overall acceptability was not significantly different (*p *>* *0.05) for all the developed yogurts, although there were differences in rating for individual attributes. The yogurt developed from the combined samples of water yam starches of *Akaba* and *Matches* had higher sensory scores in a range of 5.4–5.8 compared to the single variety samples except for 0.5% *Matches* and 1.5% *Matches* samples.

**Table 3:** Sensory scores for yogurt thickened with water yam starch

Sample	Taste	Mouthfeel	Flavor	Sourness	Consistency	Acceptance
Control	4.0 ± 1.8^ab^	4.7 ± 1.4^a^	4.3 ± 1.9^a^	3.9 ± 1.7^a^	5.3 ± 0.9^a^	4.7 ± 1.4^a^
0.5% *Akaba*	4.5 ± 1.8^ab^	5.0 ± 1.0^a^	4.4 ± 3.9^a^	4.3 ± 1.4^a^	5.0 ± 1.1^a^	4.9 ± 1.4^a^
1% *Akaba*	4.9 ± 1.4^ab^	4.5 ± 1.2^a^	4.1 ± 1.7^a^	3.8 ± 1.1^a^	4.7 ± 1.4^a^	4.5 ± 1.0^a^
1.5% *Akaba*	3.3 ± 2.0^a^	5.0 ± 0.9^a^	4.4 ± 1.4^a^	4.0 ± 1.5^a^	5.5 ± 1.2^a^	4.8 ± 1.7^a^
0.5% *Matches*	6.1 ± 1.0^b^	4.5 ± 1.3^a^	4.7 ± 1.4^a^	4.3 ± 1.7^a^	5.4 ± 1.2^a^	5.2 ± 0.9^a^
1.0% *Matches*	4.0 ± 1.6^ab^	3.8 ± 1.5^a^	3.7 ± 1.6^a^	3.6 ± 1.9^a^	4.7 ± 1.1^a^	4.2 ± 1.5^a^
1.5% *Matches*	6.4 ± 0.7^b^	5.4 ± 1.2^a^	4.9 ± 1.7^a^	4.6 ± 1.4^a^	5.5 ± 1.0^a^	5.3 ± 1.7^a^
0.5% *Akaba *+ 0.5% *Matches*	5.5 ± 1.1^ab^	5.1 ± 1.4^a^	5.4 ± 1.0^a^	4.4 ± 1.4^a^	5.6 ± 1.0^a^	5.8 ± 0.6^a^
1% *Akaba *+ 1% *Matches*	5.5 ± 1.9^ab^	4.8 ± 2.0^a^	4.9 ± 1.7^a^	4.3 ± 1.4^a^	5.9 ± 1.1^a^	5.4 ± 1.7^a^
1.5% *Akaba *+ 1.5% *Matches*	4.3 ± 1.3^ab^	5.0 ± 1.5^a^	5.3 ± 0.9^a^	4.5 ± 1.3^a^	5.5 ± 0.8^a^	5.5 ± 0.8^a^

Note

Means bearing different superscripts are significantly different (*p *<* *0.05).

Generally, clear trends were established for all the attributes of the developed yogurt samples compared to the control yogurt and the general overall acceptability for all the developed yogurts products was that of “like moderately” or better, in respect of the 7‐point Hedonic scale used in the study (Lawless & Heymann, 2010).

## 4 CONCLUSION

Water yam starches of *Akaba* variety had lower moisture content compared to the *Matches* variety. The water activity of the two starches was lower, therefore, supported the quality and shelf life of the water yam starches, which were generally low acidic. Additionally, the starches were restricted in their WAC. The SP of the starches was quite restrictive in their behavior but predominantly was lightness in color, which is a plus for new product development. The yogurt samples with combinations of water yam starches from *Akaba* plus Matches were sensory accepted more than the single variety yogurt samples. The best‐bet yogurt sample was 0.5% *Akaba *+ 0.5% *Matches,* with overall acceptability (5.8) higher than the control yogurt (4.7). This study established that water yam starches could be employed to thicken yogurts to produce transparent, creamy texture, sweet taste, flavor, consistency, and acceptable product.

## CONFLICT OF INTEREST

The authors declare that they do not have any conflict of interest.

## ETHICAL REVIEW

The study's protocols and procedures were ethically reviewed and approved by the Research and Development Committee of the CSIR‐FRI, Accra.

## INFORMED CONSENT

Written informed consent was obtained from all sensory participants.
